# The effects of *Clostridium butyricum* on Ira rabbit growth performance, cecal microbiota and plasma metabolome

**DOI:** 10.3389/fmicb.2022.974337

**Published:** 2022-09-29

**Authors:** Xiao Xing Ye, Ke Yao Li, Ya Fei Li, Jia Ning Lu, Ping Ting Guo, Hao Yu Liu, Li Wen Zhou, Shuai Shuai Xue, Cai Yun Huang, Shao Ming Fang, Qian Fu Gan

**Affiliations:** College of Animal Science (College of Bee Science), Fujian Agriculture and Forestry University, Fuzhou, China

**Keywords:** *C. butyricum*, Ira rabbits, growth performance, gut microbiota, plasma metabolites

## Abstract

Clostridium butyricum (C. butyricum) can provide many benefits for animals’ growth performance and gut health. In this study, we investigated the effects of *C. butyricum* on the growth performance, cecal microbiota, and plasma metabolome in Ira rabbits. A total of 216 Ira rabbits at 32 days of age were randomly assigned to four treatments supplemented with basal diets containing 0 (CG), 200 (LC), 400 (MC), and 600 mg/kg (HC) *C. butyricum* for 35 days, respectively. In comparison with the CG group, *C. butyricum* supplementation significantly improved the average daily gain (ADG) and feed conversion rate (FCR) at 53 and 67 days of age (*P* < 0.05) and digestibilities of crude protein (CP) and crude fiber (CF) at 67 days of age (*P* < 0.05). The cellulase activity in the HC group was higher respectively by 50.14 and 90.13% at 53 and 67 days of age, than those in the CG groups (*P* < 0.05). Moreover, at 67 days of age, the diet supplemented with *C. butyricum* significantly increased the relative abundance of Verrucomicrobia at the phylum level (*P* < 0.05). Meanwhile, the concentrations of different metabolites, such as amino acids and purine, were significantly altered by *C. butyricum* (*P* < 0.05). In addition, 10 different genera were highly correlated with 52 different metabolites at 53-day-old and 6 different genera were highly correlated with 18 different metabolites at 67-day-old Ira rabbits. These findings indicated that the *C. butyricum* supplementation could significantly improve the growth performance by modifying the cecal microbiota structure and plasma metabolome of weaned Ira rabbits.

## Introduction

Ira Rabbit is an excellent breed of meat rabbit, which has the characteristics of a high feed utilization rate and fast growth rate (Chipo and Tapiwa, 2019). In rabbit production, antibiotics as feed additives can relieve weaning stress, which can reduce huge economic losses (Hu et al., 2018). However, the long-term abuse of antibiotics in production has caused negative effects such as dysbacteriosis in animals. Therefore, it is imperative to find safe and effective alternatives to antibiotics (Alayande et al., 2020).

*Clostridium butyricum* is an acid-resisting, high temperature-resisting probiotic and can tolerate the tough intestinal environment (Chen et al., 2020; Gao et al., 2021), and non-toxigenic strains are currently used as probiotics in Asia (Cato, 1986). Butyric acid is one of the most important metabolites of *C. butyricum*, which can not only promote development but also maintain the energy source of intestinal epithelial cells (Zhang et al., 2018; Han et al., 2020). Other metabolites such as teichoic acid can promote the colonization of *C. butyricum* due to its highly adhesive properties (D’Elia et al., 2009). Meanwhile, teichoic acid can also inhibit the adhesion of Escherichia coli and protect the stability of the gut microbial structure (Gao et al., 2012). *C. butyricum* can secrete various enzymatic active substances such as cellulase, pectinase, amylase, lipase, and protease, thereby degrading the nutrients in the feed and promoting the digestion and absorption of nutrients in the intestinal tract (Zhang et al., 2016). *C. butyricum* has been proved to promote growth performance, and gut health (Hsiao et al., 2021; Liang et al., 2021) for aquatic animals (Tran et al., 2020; Luo et al., 2021), poultry (Molnár et al., 2020), and livestock (Han et al., 2020; Lopez et al., 2021). A previous study on weaning Rex rabbits has indicated that dietary supplementation with *C. butyricum* in the quantity of spore state of 1.0 × 10^5^ CFU/g increased the average daily gain and small intestinal digestive enzyme activity (Liu et al., 2019b). A basal diet supplemented with a dosage of 2.5 × 10^9^ CFU/kg *C. butyricum* can increase apparent nutrient digestibilities, and promote the proliferation of beneficial bacteria in weaned pigs, such as *Streptococcus* and *Bifidobacterium* (Han et al., 2020). Furthermore, studies have shown that *C. butyricum* MIYAIRI 588 reduced host diarrhea rate by enhancing gut colonization resistance to *Clostridioides difficile* (Hagihara et al., 2021). The intestinal microbiome is extremely important for the digestion and absorption of nutrients in animals (Turnbaugh et al., 2006; Anhê et al., 2015). However, the effect of *C. butyricum* on the growth performance and intestinal flora of the Ira rabbit is still unclear.

This study evaluated the effect of dietary supplementation of *C. butyricum* on growth performance, nutrient digestibilities of Ira rabbits. Meanwhile, the effect of *C. butyricum* on the cecal microbiota and plasma metabolites in Ira rabbits was determined. Furthermore, correlation analysis among growth performance, gut microbiome, and plasma metabolites was performed to verify the effect of *C. butyricum* on the intrinsic relationship between host and microbial metabolisms. The purpose of this study was to provide a scientific basis for the application of *C. butyricum* to partly replace antibiotics in the production of meat rabbits.

## Materials and methods

### Animal feeding experiment

A total of 216 healthy male Ira rabbits at 32 days of age with similar weight mass were randomly assigned to different single cages and raised to 67 days of age. All rabbits were randomly assigned into four groups and each group has fifty-four rabbits. The control group (CG) was fed a basal diet without the addition of *C. butyricum*, while the low dose group (LC), the medium dose group (MC), and the high dose group (HC) were fed a test diet supplemented with 200, 400 and 600 mg/kg *C. butyricum* in the basal diet, respectively. The number of viable *C. butyricum* is 2 × 10^8^ CFU/g (Hubei Lvxue Biotechnology Co., Ltd.). The feeding amount is based on the feeding test data in the internship stage, and the feeding amount is different on different days. The additional amount of Clostridium butyrate is based on the conclusion of the product description and previous experiments. The experiment was maintained for five weeks, while a 4-day pre-trail was conducted for dietary adaptation of rabbits. The basal diet met the Nutrition Research Council (NRC) for weaned rabbits and was chemically analyzed (Cunniff et al., 1995), shown in Supplementary Table 1 (Nutrition Research Council, 1930). Every cage was cleaned and disinfected by a high-temperature spray gun 3 days before rabbits were placed in them. The inner temperature was maintained at 28°C during the whole experimental period. Rabbits were exposed to continuous light from 7 a.m. to 7 p.m. per day. The rabbits in all cages were vaccinated with maternal immunity.

### Growth performance, nutrient digestibilities, and cellulase activity assay

The body weight (BW) was recorded, and the average daily gain (ADG) and feed conversion rate (FCR) were calculated at 32, 39, 46, 53, 60, and 67 days of age in Ira rabbits, respectively. The formula for FCR is:


$$F\,C\,R=\frac{\mathrm{Food}\,\mathrm{intake}\,\left(g\right)}{\mathrm{Gain}\,\mathrm{weight}\,\left(g\right)}$$

Using the total feces collection method, feces of each rabbit at 51, 52, 53 days, and 65, 66, and 67 days of age were collected, weighed, and mixed in groups. 10% of the total amount of feces, soaked in 10% sulfuric acid overnight, air-dried at 65°C for 24 h, regained moisture for 24 h, and passed through a sieve (60 mesh screen) for digestibility testing. The content of crude fiber (CF) and crude protein (CP) in diets and feces is strictly carried out following the analysis methods in “Feed Analysis and Feed Quality Testing Technology” (Zhang, 2007). The calculation formula of apparent digestibility is:


A⁢p⁢p⁢a⁢r⁢e⁢n⁢t⁢d⁢i⁢g⁢e⁢s⁢t⁢i⁢b⁢i⁢l⁢i⁢t⁢y=

$$\left(1-\frac{\mathrm{the}\,\mathrm{content}\,\mathrm{of}\,a\,\mathrm{nutrient}\,\mathrm{in}\,\mathrm{feces}}{\mathrm{the}\,\mathrm{content}\,\mathrm{of}\,a\,\mathrm{nutrient}\,\mathrm{in}\,\mathrm{feed}}\right)\times 100\%$$

At 53 and 67 days of age, 6 rabbits in every group were randomly selected before feeding, separately. The cecum aseptically and collected 3–5 g of cecal contents in the EP tube, stored at –80°C for 16s rDNA sequencing and cellulase activity detection. According to the manufacturer’s instructions, the kit (Nanjing Jiancheng Institute of Bioengineering) was used to determine cellulase activity. Blood was collected from the ear vein, centrifuged at 3500 r/min for 10 min, and the supernatant was taken and stored at –80°C for plasma metabolomics test.

### 16S rDNA sequencing analysis

According to the manufacturer’s instructions, microbial community genomic DNA was extracted from cecal content samples using the E.Z.N.A.^®^ soil DNA Kit (Omega Bio-Tek, Norcross, GA, USA). The hypervariable region V3-V4 of the bacterial 16S rDNA was amplified with primer pairs 338F (5′-ACTCCTACGGGAGGCAGCAG-3′) and 806R (5′-GGACTACHVGGGTWTCTAAT-3′) by an ABI GeneAmp^®^ 9700 PCR thermocycler (ABI, CA, USA). The purified products of amplification were sequenced on the Hiseq-2500 platform (Illumina, USA). The quality control of the original data was completed by QIIME (QIIME 1.9.1, USA). The raw 16S rDNA sequencing reads were demultiplexed and quality-filtered by fastp version 0.20.0 (Chen et al., 2018) and merged by FLASH version 1.2.7 (Magoč and Salzberg, 2011) with the following criteria: (i) the 300 bp reads were truncated at any site receiving an average quality score of < 20 over a 50 bp sliding window, and the truncated reads shorter than 50 bp were discarded, reads containing ambiguous characters were also discarded; (ii) only overlapping sequences longer than 10 bp were assembled according to their overlapped sequence. The maximum mismatch ratio of the overlap region is 0.2. Reads that could not be assembled were discarded; (iii) Samples were distinguished according to the barcode and primers, and the sequence direction was adjusted, exact barcode matching, 2 nucleotide mismatch in primer matching. Operational taxonomic units (OTUs) with 97% similarity cutoff were clustered using UPARSE version 7.1 (Stackebrandt and Goebel, 1994; Edgar, 2013), and chimeric sequences were identified and removed. The taxonomy of each OTU representative sequence was analyzed by RDP Classifier version 2.2 (Wang et al., 2007) against the 16S rDNA database (e.g., Silva v138) using a confidence threshold of 0.7. The alpha and beta diversity indices were calculated using QIIME (v.1.9.1). Beta diversity was evaluated by principal component analysis (PCA) plots based on Euclidean distances using the R. Bar plot was constructed to analyze the microbial composition at the phylum and genus levels based on OTU abundance using the R package (animalcules).

### LC-MS analysis

A 100 μL plasma sample was transferred to an E.P. tube, and 400 μL extract solution (acetonitrile: methanol = 1: 1) containing internal standard (L-2-Chlorophenylalanine, 2 μg/mL) was added. After 30 s vortex, the samples were sonicated for 10 min in the ice-water bath. Then the samples were incubated at –40°C for 1 h and centrifuged at 10,000 rpm for 15 min at 4°C. 400 μL supernatant was transferred to a fresh tube and dried in a vacuum concentrator at 37°C. Then, the dried samples were reconstituted in 200 μL 50% acetonitrile by sonication on ice for 10 min. The solution was centrifuged at 13000 rpm for 15 min at 4°C, and 75 μL of supernatant was transferred to a fresh glass vial for LC/MS analysis. The quality control (QC) sample was prepared by mixing an equal aliquot of the supernatants from all samples. The UHPLC separation was carried out using a 1290 Infinity series UHPLC System (Agilent Technologies Co., Ltd., Los Angeles, USA), equipped with a UPLC BEH Amide column (2.1 * 100 mm, 1.7 μm, Waters, Shanghai, China). The mobile phase consisted of 25 mmol/L ammonium acetate and 25 mmol/L ammonia hydroxide in water (pH = 9.75) (A) and acetonitrile (B). The analysis was carried with elution gradient as follows: 0∼0.5 min, 95% B; 0.5∼7.0 min, 95∼65% B; 7.0∼8.0 min, 65∼40% B; 8.0∼9.0 min, 40% B; 9.0∼9.1 min, 40∼95% B; 9.1∼12.0 min, 95% B. The column temperature was 25°C. The auto-sampler temperature was 4°C, and the injection volumes were always 2 μL (positive ion mode) or 2 μL (negative ion mode) (Wang et al., 2016), respectively. The TripleTOF 6600 mass spectrometry (AB Sciex) was used for its ability to acquire MS/MS spectra on an information-dependent basis (IDA) during an LC/MS experiment. In this mode, the acquisition software (Analyst TF 1.7, AB Sciex) continuously evaluates the full scan survey M.S. data as it collects and triggers the acquisition of MS/MS spectra depending on preselected criteria. The most intensive 12 precursor ions (intensity > 100) were chosen for MS/MS at collision energy (CE) of 30 eV in each cycle. The cycle time was 0.56 s. The ESI source conditions were set as follows: Gas 1 as 60 psi, Gas 2 as 60 psi, Curtain Gas as 35 psi, Source Temperature as 600°C, Declustering potential as 60 V, Ion Spray Voltage Floating (ISVF) as 5000 V or –4000 V in positive or negative modes, respectively.

### Metabolomics data analysis

MS raw data (.wiff) files were converted to the mzXML format by ProteoWizard and processed by the R package XCMS (version 3.2). The process includes peak deconvolution, alignment, and integration. Minfrac and cut-off were set as 0.5 and 0.3, respectively. An in-house MS2 database was applied for metabolite identification (Smith et al., 2006). After obtaining the sorted data, we conduct a series of multivariate pattern recognition analyses on it. The first is principal component analysis. Wilcoxon rank-sum test with false discovery rate (FDR) correction was performed to detect differences in microbial diversity indices and relative abundances of microbes at different taxonomic levels. Correlations between different metabolites and bacterial communities were assessed by Spearman’s correlation analysis using the pheatmap package in R (Kolde, 2015). FDR adjusted *P*-values and the corrected *P*-values below 0.05 were statistically significant.

### Statistical analysis method

The results of this study are presented as the mean ± standard deviation, and a one-way analysis of variance (One-way ANOVA) was performed on the test results using GraphPad Prism8 software, and Turkey multiple comparison was used. *P* < 0.05 means significant difference, *P* < 0.01 means extremely significant difference.

## Results

### Growth performance

The addition of *C. butyricum* significantly affected the BW, ADG, and FCR for Ira rabbits (Table 1). At 53 and 67 days of age, the BW of the HC and MC groups were significantly increased by 4 and 3.35% (*P* < 0.05) compared with CG, and the ADG of HC and LC groups were significantly increased by 7.92 and 4.65% (*P* < 0.05) compared with CG. The MC group performed best in FCR at 53 days of age, which was 8.75% better than the CG group (*P* < 0.05). At 67 days of age, the FCR of the HC group was significantly better than that of the CG group (*P* < 0.05).

**TABLE 1:** The effects of *C. butyricum* on the growth performance of Ira rabbits.

Indexes	Days of Age	CG	LC	MC	HC
Initial BW (kg)	32	0.79 ± 0.10	0.91 ± 0.99	0.79 ± 0.08	0.79 ± 0.09
BW (kg)	53	1.75 ± 0.15^c^	1.76 ± 0.02^c^	1.79 ± 0.02^b^	1.82 ± 0.08^a^
ADG (g)		44.2 ± 0.50^c^	46.3 ± 2.30^b^	45.6 ± 2.20^c^	47.7 ± 3.80^a^
Feed intake (g)		8.8526	9.2661	7.3736	7.8256
FCR		2.97 ± 0.33^ab^	3.04 ± 0.42^a^	2.71 ± 0.14^c^	2.79 ± 0.13^bc^
BW (kg)	67	2.39 ± 0.07^b^	2.38 ± 0.11^b^	2.31 ± 0.07^b^	2.47 ± 0.11^a^
ADG (g)		45.2 ± 0.55^b^	44.5 ± 5.40^b^	43.3 ± 4.20^b^	47.3 ± 3.40^a^
Feed intake (kg)		6.5305	6.3359	5.4558	4.9665
FCR		3.44 ± 0.42^a^	3.39 ± 0.44^a^	3.00 ± 0.28^ab^	2.50 ± 0.14^b^

Means with different superscript letters within the same line differ significantly, *P* < 0.05; data are presented as mean ± SEM (*n* = 6). BW, body weight; ADG, average daily gain; FCR, feed conversion ratio.

### Nutrients digestibilities and cellulase activity in Ira rabbit cecum

The addition of *C. butyricum* significantly affected the digestibility of CP and CF (Table 2). At 53 days of age, the digestibility of CF in the HC group was significantly higher than in the CG group (*P* < 0.05). At 67 days of age, the digestibilities of CP and CF in the HC group were significantly higher than in the CG group (*P* < 0.05). Moreover, at 53 and 67 days of age, the cellulase activity in the HC group was significantly increased by 50.14 and 90.14% compared with the CG group (*P* < 0.05, Table 3).

**TABLE 2:** The effects of *C. butyricum* on nutrients digestibilities in Ira rabbit cecum.

Digestibilities, %	Days of Age	CG	LC	MC	HC
CP	53	80.10 ± 2.58	80.79 ± 4.09	81.79 ± 4.10	84.46 ± 4.21
CF		46.96 ± 2.61^b^	49.15 ± 1.47^b^	53.67 ± 2.62^a^	55.26 ± 2.30^a^
CP	67	77.97 ± 2.47^b^	78.87 ± 1.41^b^	80.43 ± 1.14^a^	81.46 ± 2.40^a^
CF		43.57 ± 2.55^b^	45.13 ± 2.30^b^	45.97 ± 1.15^b^	50.35 ± 1.40^a^

Means with different superscript letters within the same line differ significantly, *P* < 0.05; data are presented as mean ± SEM (*n* = 6). CP, crude protein; CF, crude fiber.

**TABLE 3:** The effect of *C. butyricum* on cellulase activity in Ira rabbit cecum.

Index, %	Days of Age	CG	LC	MC	HC
Cellulase activity	53	17.35 ± 1.21^b^	20.95 ± 4.00^ab^	24.44 ± 9.60^ab^	26.05 ± 3.74^a^
	67	16.02 ± 0.92^b^	23.86 ± 3.72^ab^	27.65 ± 4.60^a^	30.46 ± 1.57^a^

Means with different superscript letters within the same line differ significantly, *P* < 0.05; data are presented as mean ± SEM (*n* = 6).

### Structure and diversity of cecal microbiota in Ira rabbits at different stages

**FIGURE 1:**
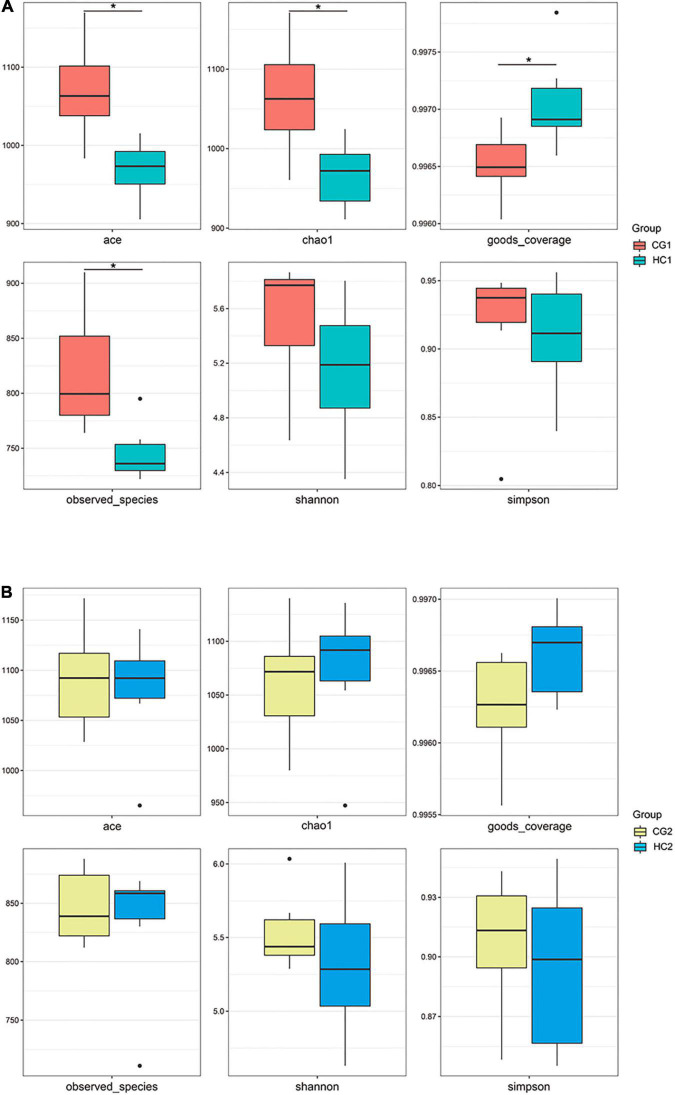
Changes in Alpha diversity index of cecal microbiota after *C. butyricum* supplementation at 53 **(A)** and 67 **(B)** days of age. CG1, control group at 53 days of age; CG2, control group at 67 days of age; HC1, the high dose at 53 days of age; HC2, the high dose at 67 days of age. *P < 0.05, **P < 0.01, ***P < 0.0001, and ****P < 0.0001.

**FIGURE 2:**
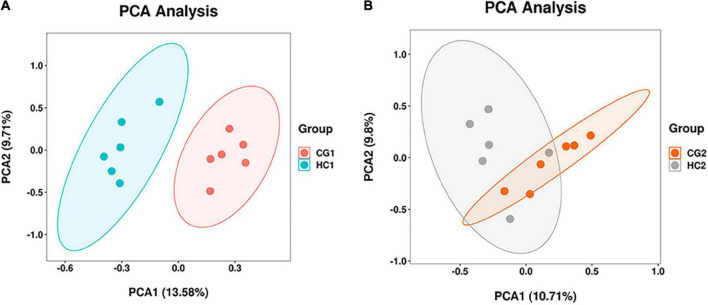
PCA of cecal microbiota at 53 **(A)** and 67 **(B)** days of age. CG1, control group at 53 days of age; CG2, control group at 67 days of age; HC1, the high dose at 53 days of age; HC2, the high dose at 67 days of age.

A total of 2,472,383 tags in 24 rabbits (two groups for two sampling times) were obtained according to the quality-controlled 16S rDNA gene sequencing. At the age of 53 days, the ace, chao1, and observed species indexes of the HC1 group showed a significant upward trend compared with the CG1 group, while the goods_coverage showed a significant downward trend. At 67 days of age, there were no significant differences (*P* > 0.05) between the estimates of alpha diversity index (Figure 1). PCA was carried out to study the composition distance relationship using dimensionality reduction. The intestinal bacteria in each group were separated, represented by the group distances (Figure 2).

**FIGURE 3:**
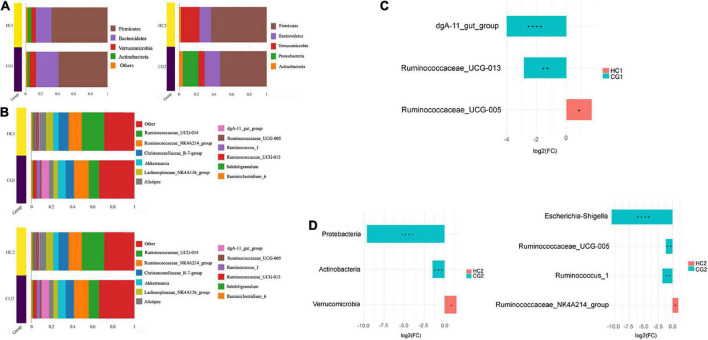
The relative abundance of cecal microbiota in Ira rabbits. **(A)** Phylum level. **(B)** Genus level. **(C)** Between-group variance analysis of genus at 53 days of age. **(D)** Between-group variance analysis of phylum and genus at 67 days of age. CG1, control group at 53 days of age; CG2, control group at 67 days of age; HC1, the high dose at 53 days of age; HC2, the high dose at 67 days of age. In the figure, * means *P* < 0.05, ** means *P* < 0.01, *** means *P* < 0.0001, **** means *P* < 0.0001, the same as in the figure below.

At the phylum level (Figure 3A), Firmicutes and Bacteroidetes were the top two predominant phyla in each group, containing 59.43% (CG1), 59.15% (CG2), 68.59% (HC1), and 68.22% (HC2), respectively. Meanwhile, the Firmicutes, Bacteroidetes, Verrucomicrobia, and Actinobacteria counted for over 95% of the gut microbiota (Figure 3A). At the genus level, *Ruminococcaceae NK4A214 group*, *Ruminococcaceae UCG-014*, *Akkermansia*, *Ruminococcaceae UCG-013*, *Christensenellaceae R-7 group*, and *Lachnospiraceae NK4A136 group* were the predominant genera (Figure 3B). Furthermore, the differential analysis [Log2(FC)] between each group among phylum and genus levels was analyzed as well. At 53 days of age, the relative abundance of *dgA-11 gut group*, *Ruminococcus UCG-013* significantly decreased, however, the *Ruminococcus UCG-005* increased in the HC1 group (*P* < 0.05, Figure 3C). At 67 days of age, the relative abundances of *Escherichia-Shigella*, *Ruminococcus UCG-005*, and *Ruminococcus 1* significantly increased, while *Ruminococcaceae NK4A214 group* significantly increased in the HG2 group (*P* < 0.05, Figure 3D).

### Plasma metabolomics

**FIGURE 4:**
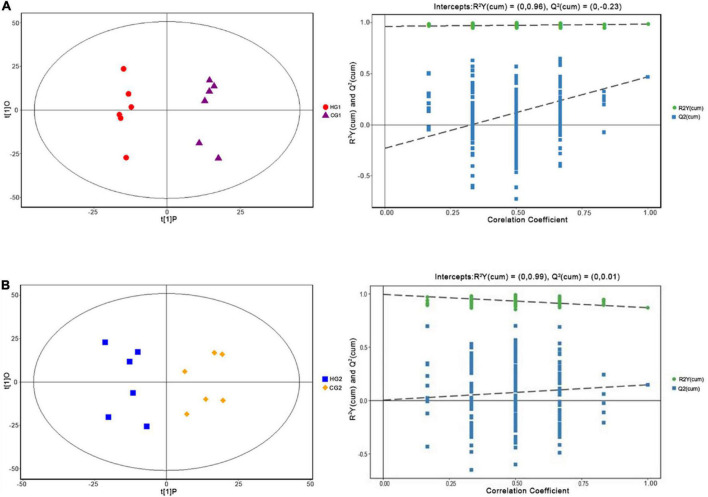
Orthogonal partial least squares discriminant analysis (OPLS-DA) plot of plasma metabolites of Ira rabbits supplied with *C. butyricum* at 53 days of age **(A)** and 67 days of age **(B)**.

Metabolomic analysis was conducted to explore the effect of *C. butyricum* on plasma metabolic profiles. In this study, 1,897 peaks were detected in the positive level, and 1,875 metabolites were left after relative standard deviation de-noising, while 1,850 metabolites were in the negative level (Supplementary Table 2). The OPLS-DA score plot and permutation test further showed a significant difference between the two stages (Figures 4A,B), suggesting that *C. butyricum* caused metabolic phenotype alterations in rabbits’ plasma. The OPLS-DA model was used to determine the differential metabolites between the pairwise comparison groups with the first principal component of variable importance in projection (VIP) values (VIP > 1) combined with *P* < 0.05 (Supplementary Table 3).

**FIGURE 5:**
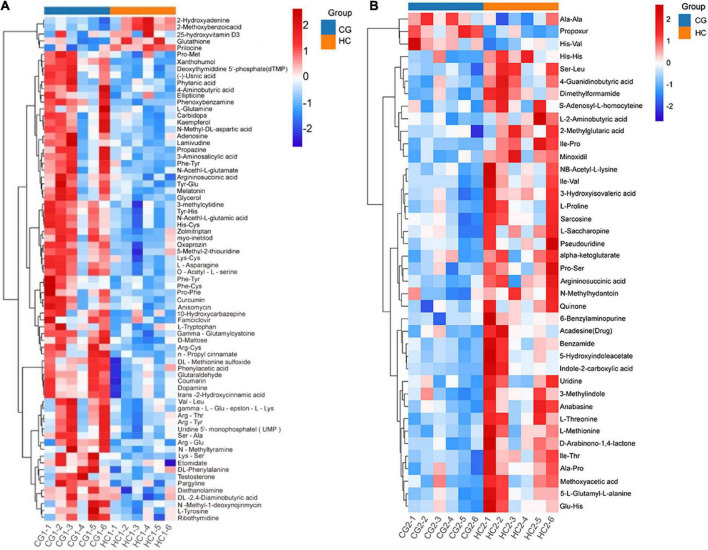
Hierarchical clustering analysis for plasma metabolites in Ira rabbits in the high dose at 53 days of age **(A)** and 67 days of age **(B)**. Each column in the figure represents a sample.

**FIGURE 6:**
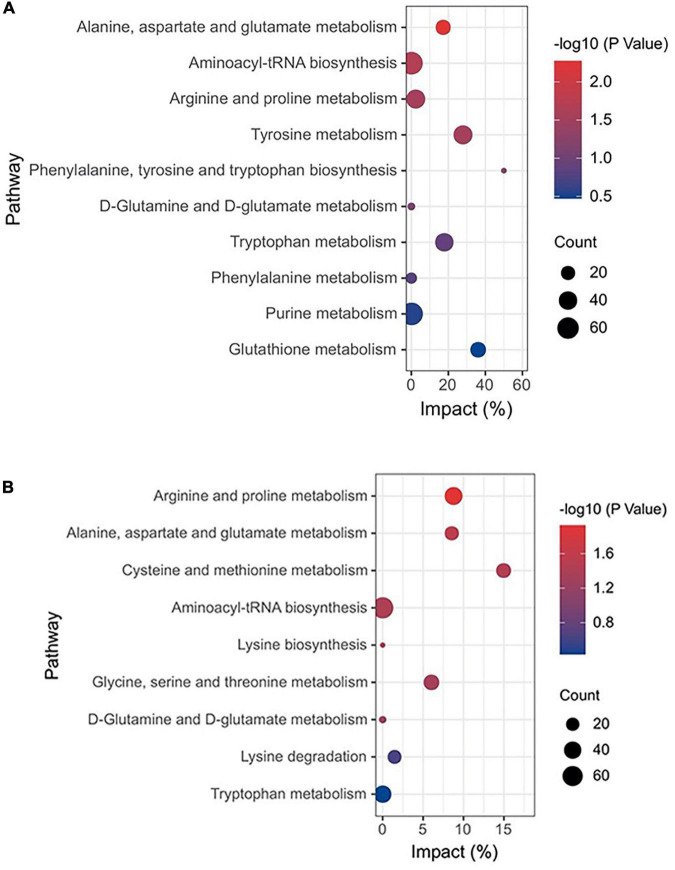
KEGG function annotation analysis of plasma metabolites in Ira rabbits. **(A)** The bubble plot of KEGG indicates different enrichments affected by *C. butyricum* of Ira rabbits at 53 days of age. **(B)** The bubble plot of KEGG indicates different enrichments affected by *C. butyricum* of Ira rabbits at 67 days of age.

Heatmap described the differential metabolites between the CG and HC groups of two stages. Compared with the CG, the HC contained 5 upregulated and 69 downregulated differential metabolites at 53 days of age, and 37 upregulated and 3 downregulated differential metabolites at 67 days of age (Figures 5A,B). To explore the potential metabolic pathways affected by *C. butyricum*, all the differential metabolites were further analyzed by KEGG annotation. At 53 days of age, compared with the CG, the most enriched pathways in the HC were “Alanine, aspartate, and glutamate metabolism,” “Aminoacyl-tRNA biosynthesis,” “Arginine and proline metabolism,” “Tyrosine metabolism,” and “phenylalanine, tyrosine, and tryptophan biosynthesis” (Figure 6A). At 67 days of age, compared with the CG, the most enriched pathways in the HC were “Arginine and proline metabolism,” “Alanine, aspartate and glutamate metabolism,” “Cysteine and methionine metabolism,” “Aminoacyl-tRNA biosynthesis,” and “Lysine biosynthesis” (Figure 6B).

### Correlation analysis between growth performance and plasma metabolites

**FIGURE 7:**
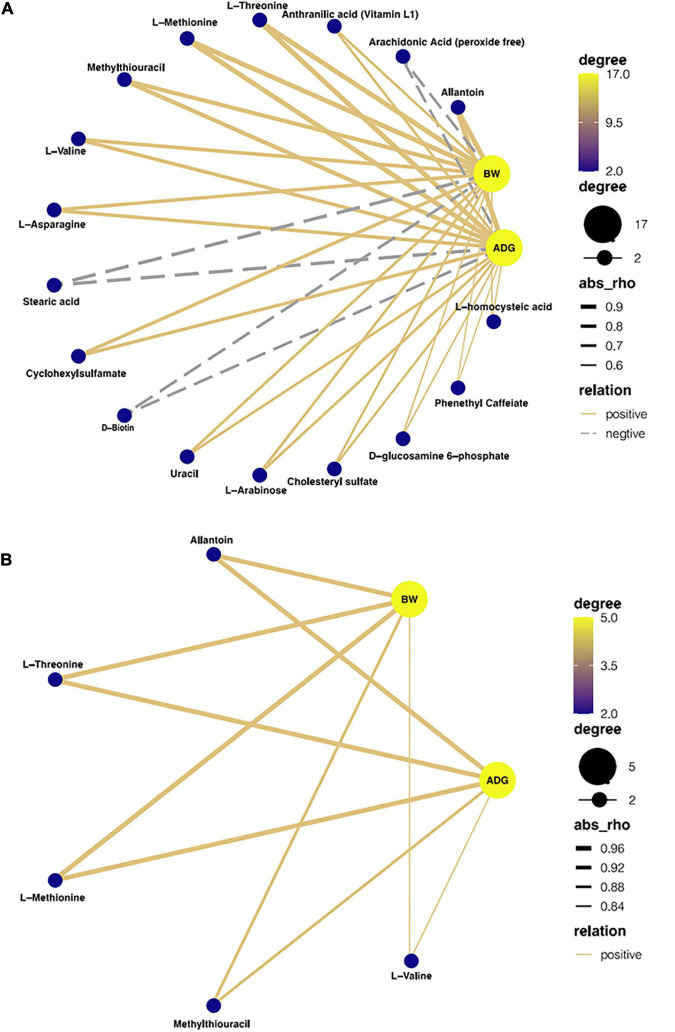
Interaction network analysis between growth performance and metabolites in Ira rabbits. **(A)** 53 days old; **(B)** 67 days old. Blue nodes represent the center degree of genera and metabolites. The node size indicates the level of center degree. Lines linked to nodes indicate significant correlations among the species (FDR adjusted *P* < 0.05, | r| > 0.5), with orange and gray colors showing positive and negative correlations, respectively.

According to Spearman’s correlation coefficient analysis, there were correlations between BW, ADG, and different metabolites. From Figure 7A, at 53 days of age, L-Valine, L-Methionine, L-Asparagine, L-Threonine, Allantoin, Anthranilic acid (Vitamin L1), Methylthiouracil, Cyclohexylsulfanmate, Uracil, L-Arabinose, Cyclohexylsulfamate, D-glucosamine 6-phosphate, Phenethyl Caffeiate, and L-homocysteic acid showed a positive correlation with BW and ADG. Arachidonic Acid (peroxide-free), Stearic Acid, and D-Biotin were negatively correlated with BW and ADG. From Figure 7B, at 67 days of age, Allantoin, L-Threoine, L-Methionine, Methylthiouracil, and L-Valine were positively correlated with BW and ADG.

### Correlation analysis between gut microbiota and plasma metabolites

**FIGURE 8:**
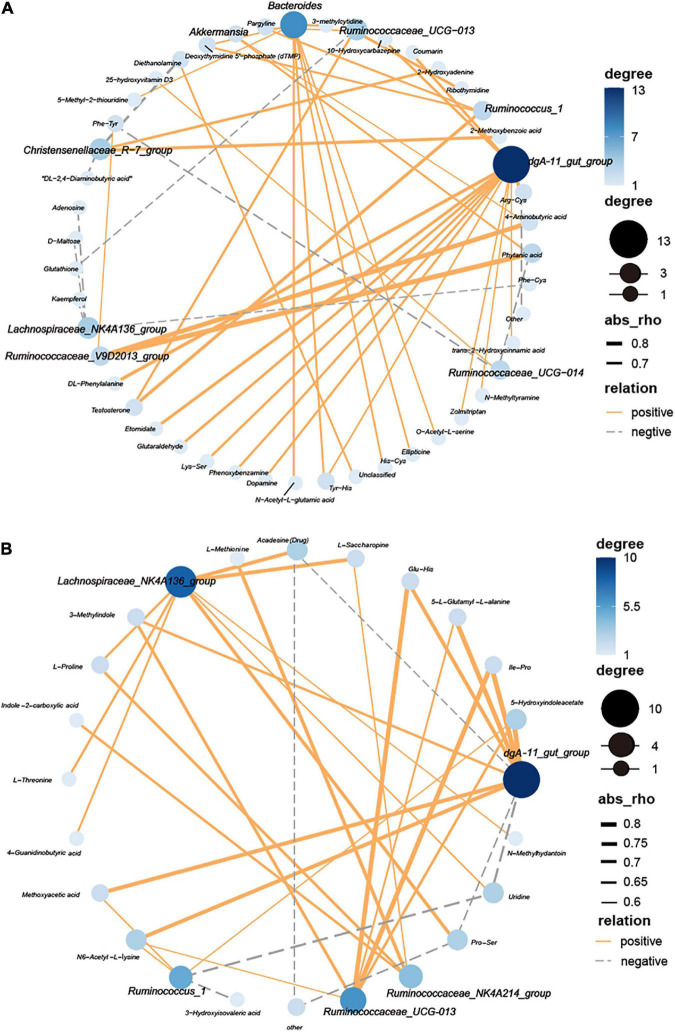
Interaction network analysis between cecal bacteria (bold font) and plasma metabolites of Ira rabbits. **(A)** 53 days of age. **(B)** 67 days of age. Blue nodes represent the center degree of genera and metabolites. The node size indicates the level of center degree. Lines linked to nodes indicate significant correlations among the species (FDR adjusted *P* < 0.05, | r| > 0.5), with orange and gray colors showing positive and negative correlations, respectively.

To reveal the relationships between gut microbial (at the genera level) and the different metabolites, network Diagram were generated by Spearman correlation analysis (Figure 8). Ten critical genera were highly correlated with 52 different metabolites (| r| > 0.5, FDR adjusted *P* < 0.05) at 53 days old (Figure 8A). Moreover, six important genera were highly correlated with 18 significant metabolites (| r| > 0.6, FDR adjusted *P* < 0.05) at 67 days old (Figure 8B).

## Discussion

Increasing evidence suggested the opinion that *C. butyricum* can enhance gut health and animal growth (Huang et al., 2021; Li et al., 2021a,b; Yu et al., 2021). Meanwhile, emerging studies of gut microbial and metabolome correlation linked to the growth performance, healthy, and development of livestock (Park et al., 2013; Tian et al., 2015; Huang et al., 2019). Therefore, the understanding of gut microbial and plasma metabolome of Ira rabbits can reveal the possibility of improving the growth performance of rabbits. However, few studies investigated the relationship between microbial and metabolome of Ira rabbits in different growth stages. Thus, we explored the differences in the gut microbial and plasma metabolome of Ira rabbits and their correlations affected by the supplementation of *C. butyricum*.

In this study, the addition of *C. butyricum* significantly promoted ADG and FCR (Table 1, *P* < 0.05) of Ira rabbits at different growth stages. Meanwhile, at 67 days of age, *C. butyricum* at a dose of 600 mg/kg in the diet significantly increased the digestibility of CF and CP (Table 2, *P* < 0.05). Several reports have shown that *C. butyricum* exhibited a significant positive influence on growth performance and nutrient utilization efficiency in animals (Han et al., 2020; Li et al., 2021a; Liu et al., 2021), consistent with the findings of this study. It was reported that the supplementation of *C. butyricum* caused an increase in the villus height and enlarged crypt depth, which possibly improve the ADG caused by the increased absorption of intestinal cells (Huang et al., 2021). In this study, cellulase activity was enhanced at different dosages of *C. butyricum*. Thus, it can be demonstrated that *C. butyricum* supplementation has positive effects on the growth performance of Ira rabbits.

As a pivotal part of the intestinal tract, the gut microbial is called the black box of bodies. All the digestibility, development, maintenance, metabolism, and immunity have related to the gut microbial for rabbits (Huang et al., 2021). We evaluated whether the supplement of *C. butyricum* in the diet could alter the microbial community structure, similar to previous results (Huang et al., 2021). We found that the abundance of Firmicutes and *Ruminococcaceae_UGG-014* were changed by the *C. butyricum* supplementation (Figures 3A,B). The Ace, Chao1, and Observed species and goods_coverage indexes, were changed by the *C. butyricum.* The results were similar to the earlier studies (Duan et al., 2018; Casas et al., 2020) on rabbits and pigs.

The homeostasis of the gut microbiota has an important influence on maintaining the body’s growth and development, nutrient digestion, and absorption (Shen et al., 2006). Our previous study found that 10.42% of the body weight change in weaned Ira rabbits could be explained by the gut microbiome (Fang et al., 2020). Therefore, the effect of *C. butyricum* on the intestinal microbiota of Ira rabbits needs to be considered. The present study showed that Firmicutes, Bacteroides, and Verrucomicrobia were the dominant phyla in weaning and fattening rabbits, consistent with previous findings (Li et al., 2018; Mattioli et al., 2019). Firmicutes play an essential role in degrading diet fiber and cellulose, which are advantageous to cellulose degradation (Zhu et al., 2015). Bacteroides can facilitate the non-digestible polysaccharides metabolism and produce essential vitamins and protein (Avershina et al., 2016; Jin et al., 2018). We found that diets supplemented with *C. butyricum* showed a trend of increasing the ratio of Firmicutes/Bacteroidetes. Meanwhile, *C. butyricum* supplementation significantly increased the relative abundance of Verrucomicrobia for rabbits at 67 days of age. Verrucomicrobia is a relatively newly defined phylum whose functions are largely unknown. However, the survival rate of fattening rabbits is primarily related to the incidence of epizootic rabbit enteropathy (Davies and Davies, 2003; Jin et al., 2018), which could explain the decrease in the relative abundance of Verrucomicrobia in healthy rabbits.

In this study, *Ruminococcaceae NK4A214 group*, *Ruminococcaceae UGG-014*, and *Akkermansia* were the dominant genera in the growing period of Ira rabbits. The *Ruminococcaceae*, as a member of Firmicutes, such as *Ruminococcaceae NK4A214 group*, *Ruminococcaceae UCG-014*, *Ruminococcaceae V9D2013 group*, and *Ruminococcaceae UCG-013*, have been reported to play a role in the fermentation of dietary fiber and polysaccharides (Louis et al., 2010; Chen et al., 2017; Zhao et al., 2017). Meanwhile, *Ruminococcaceae* one of the most abundant families of the order *Clostridiales* is associated with the maintenance of gut healthy (Biddle et al., 2013). In addition, they primarily produce butyrate in the gut (Ley et al., 2006; Louis and Flint, 2009). Butyrate generated by gut microbiota is a significant energy source for gut microbes and host colonic epithelium, which maintains the gut barrier functions (Bui et al., 2015; Riviére et al., 2016) and plays a critical role in colon health (Louis and Flint, 2009). Importantly, butyrate is recognized by G protein-coupled receptors FFAR3 and GPR109A and is involved in regulating energy and nutrient metabolism and having an anti-inflammatory effect (Le Poul et al., 2003; Ahmed et al., 2009; Kasubuchi et al., 2015). The relative abundances of *Ruminococcaceae*, which degrade solid feed, increase with age and become more dominant during the post-weaning period (Padilha et al., 1995). In this study, we investigated that the increase of the relative abundance of *Ruminococcaceae_NK4A214_group* was affected by the *C. butyricum* supplementation, which enhanced the superior position of Firmicutes in the intestinal microbial. This study found that the relative abundance of family *Lactobacillus* (*Lachnospiraceae NK4A136 group*) increased with the dose of *C. butyricum* and decreased with age, which contributes to the metabolism of breast milk (Jost et al., 2015; de Muinck and Trosvik, 2018).

Metabolomics is a new “omics” including genomics, transcriptomics, and proteomics (Liang et al., 2021). The subtle changes in metabolite content directly correlate with changes in biological phenotype (Nobeli and Thornton, 2006). To identify the *C. butyricum*-based metabolites, we investigated the characteristics of plasma metabolomics. In this study, *C. butyricum* supplementation significantly influenced the dose of metabolites belonging to fatty acids, amino acids, and organic acids (Figures 6A,B). The content of L-Valine, L-Methionine, and L-Threonine were upregulated in the HC group of *C. butyricum* (Figure 6B). Valine is an important essential amino acid for rabbits, and is cataloged as a branched-chain amino acid. After transamination, oxidative decarboxylation, and dehydrogenation, succinic monoacyl CoA is produced, which enters into the tricarboxylic acid cycle and supplies energy to bodies (Liang et al., 2021). In this study, the association analysis revealed a high correlation between L-Valine and BW, and ADG, which is consistent with these findings (Figures 7A,B). Therefore, *C. butyricum* could improve the growth performance of rabbits by promoting amino acid metabolism. L-Methionine is a precursor of other sulfur-containing amino acids and a limiting amino acid for rabbits, and it plays an essential role in protein synthesis of cecal bacteria and regulation of mucosal response to antigens as well (Firkins et al., 2015; Mariz et al., 2018; Liu et al., 2019a). L-Threonine is a major component of mucins (MUCs) and γ-globulins (Wang et al., 2009). In addition, Arginine and glutamine are the substrates to synthesize the functional amino acid L-Proline, upregulated in the experimental group (HC group) in this study. L-Proline is a significant amino acid for maintaining cell structure and functions and functioning as an essential regulator of cell metabolism and physiology. Proline is also an essential precursor in protein synthesis and anti-oxidative reactions in wounds and immune responses (McAllan and Smith, 1973; Ametaj et al., 2010). Furthermore, this study discovered increases in the concentrations of metabolites associated with purine metabolism. Purine is catabolized through a few intermediates to hypoxanthine converted to Xanthine; thus, Xanthine is a biomarker of microbial protein synthesis (Duan et al., 2017; Yiwei and Chi, 2019).

An earlier study has revealed that there was a reciprocal relationship between gut microbes and several metabolites that maintains intestinal homeostasis (Vascellari et al., 2020). The altered metabolome profile could influence the differences in the gut microbiome of animals (Coleman et al., 2019; Ye et al., 2021). In our study, several beneficial bacteria, such as families *Ruminococcaceae (Ruminococcaceae NK4A214 group)* and *Lachnospiraceae (Lachnospiraceae NK4A136 group*)were positively correlated with amino acids (e.g., L-Valine, L-Methionine, and L-Threonine), organic acids (e.g., Indole-2-carboxylic acid, 4-Guanidinobutyric acid, 3-Hydroxyisovaleric acid), and fatty acids (e.g., Methoxyacetic acid). These results suggest that, for Ira rabbits, *C. butyricum* can alter the gut microbial community and plasma metabolome, implying that the interactions among gut microbial-plasma metabolome are important. However, their mechanisms still need to be explored.

Throughout our study, *C. butyricum* was shown to directly influence the gut bacterial community and plasma metabolites in rabbits. Accumulating evidence indicates that phenotypic traits of animals are changed by gut microbes, while the concentrations of plasma metabolites influence the functions of gut bacteria to synthesize and metabolize nutrients (Henry et al., 2020; Tang et al., 2020). Overall, these changes and relationships reveal essential features associated with *C. butyricum* supplementation in rabbit diets and that alteration in the gut microbial composition and metabolic profiles may affect rabbit production efficiency.

## Conclusion

The study results suggested that, based on the different doses of *C. butyricum*, the growth performance was significantly influenced by the *C. butyricum*. We examined the overall comprehension of the patterns of microbial community and metabolite compositions at different growth stages of Ira rabbits. The microbial community was changed by the supplementation of *C. butyricum*, especially prompting the beneficial microbial, which have related to the growth performance of Ira rabbits. Meanwhile, the metabolites were affected by the *C. butyricum*. The control group and the experimental group had different contents of significant metabolites. Furthermore, Spearman’s correlation analysis revealed an obvious correlation between the microbiota and metabolites, meanwhile, a strong correlation between the metabolome and growth performance was discovered as well. Our results provide information that could aid future studies in determining the microbiome-metabolome interactions and how it influences the growth performance of Ira rabbits.

## Data availability statement

The datasets presented in this study can be found in online repositories. The names of the repository/repositories and accession number(s) can be found below: NCBI, PRJNA861087.

## Author contributions

XX contributed to conception and design of the study, performed the experiments, and revised the manuscript. KL analyzed the data and wrote and revised the manuscript. KY, JN, PT, and HY performed the experiments, analyzed the data, and revised the manuscript. LW, SS, CY, and SM performed the experiments. QF conceived and designed the experiments, supervised the experiment progress, and revised the manuscript. All authors read and approved the final manuscript.
